# A new species of frog (Terrarana, Strabomantidae, *Phrynopus*) from the Peruvian Andean grasslands

**DOI:** 10.7717/peerj.9433

**Published:** 2020-06-24

**Authors:** Germán Chávez, Luis Alberto García Ayachi, Alessandro Catenazzi

**Affiliations:** 1 División de Herpetología, CORBIDI, Lima, Perú; 2 Department of Biological Sciences, Florida International University, Miami, FL, United States of America; 3 Instituto Peruano de Herpetología, Lima, Perú

**Keywords:** *Phrynopus*, Andes, Peru, Areolate venter, Phylogenetic, Lineage, Huánuco, Marañón

## Abstract

We describe a new, medium-sized species of terrestrial frog of the genus *Phrynopus* from a single locality in the central Andes of Peru (Departamento de Huánuco) at 3,730 meters of elevation. Phylogenetic analyses supported *Phrynopus remotum* sp. nov. as an independent lineage, sister to most of its congeners. The new species is morphologically distinguishable by the presence of small tubercles on upper eyelids and heels, an areolate venter, and the absence of dorsolateral folds or ridges. This species inhabits the highlands adjacent to the Marañón Dry valley. The only sympatric amphibian species recorded is the marsupial frog *Gastrotheca peruana*.

## Introduction

Terrestrial-breeding frogs of the genus *Phrynopus* inhabit Andean highland habitats such as puna grasslands and montane forests in central and northern Peru (Lehr, Moravec & Cusi, 2012). Twelve species (over a third of the current diversity of the genus) have been described since 2012. Except for *P. mariellaleo* Venegas et al. (2018) from northern Peru, all other recently described species occur in central Peru. Moreover, the distribution ranges of most species are restricted to one or two localities, and rarely overlap with the range of congeneric species. In light of this small geographic range, it is not surprising that researchers continue to discover new species in the high Andes of central of Peru (Lehr & Rodriguez, 2017; Lehr et al., 2017).

The Andes mountain range runs through Peru in a north-south direction, and is divided into three ridges that run parallel to each other, and which only meet at the Nudo de Pasco (central Peru), and at the south in the Nudo de Vilcanota (southern Peru). The Andean topography is very rugged and its elevation can range from 100 to 4,000 m a.s.l. in a few kilometers. To reach its current forms, the Andean mountains have experienced multiple uplift events since the Miocene (Gregory-Wodzicki, 2000), which included volcano eruptions and earthquakes, resulting in an altitudinal gradient that heavily affected climatic conditions, habitats, and the diversity of the organisms inhabiting these lands. These geographical isolation events promoted vicariance, an important mechanism of vertebrate diversification and endemism in the Andes (Duellman, 1999; Wiens, 2007; Santos et al., 2009; Benham & Witt, 2016; Hazzi et al., 2018; Wollenberg-Valero et al., 2019). Mountain top isolation could also explain why *Phrynopus*, despite having a small distribution range in the Central Andes, is one of the most diverse clades among Andean highland amphibians (Frost, 2020).

**Figure 1:**
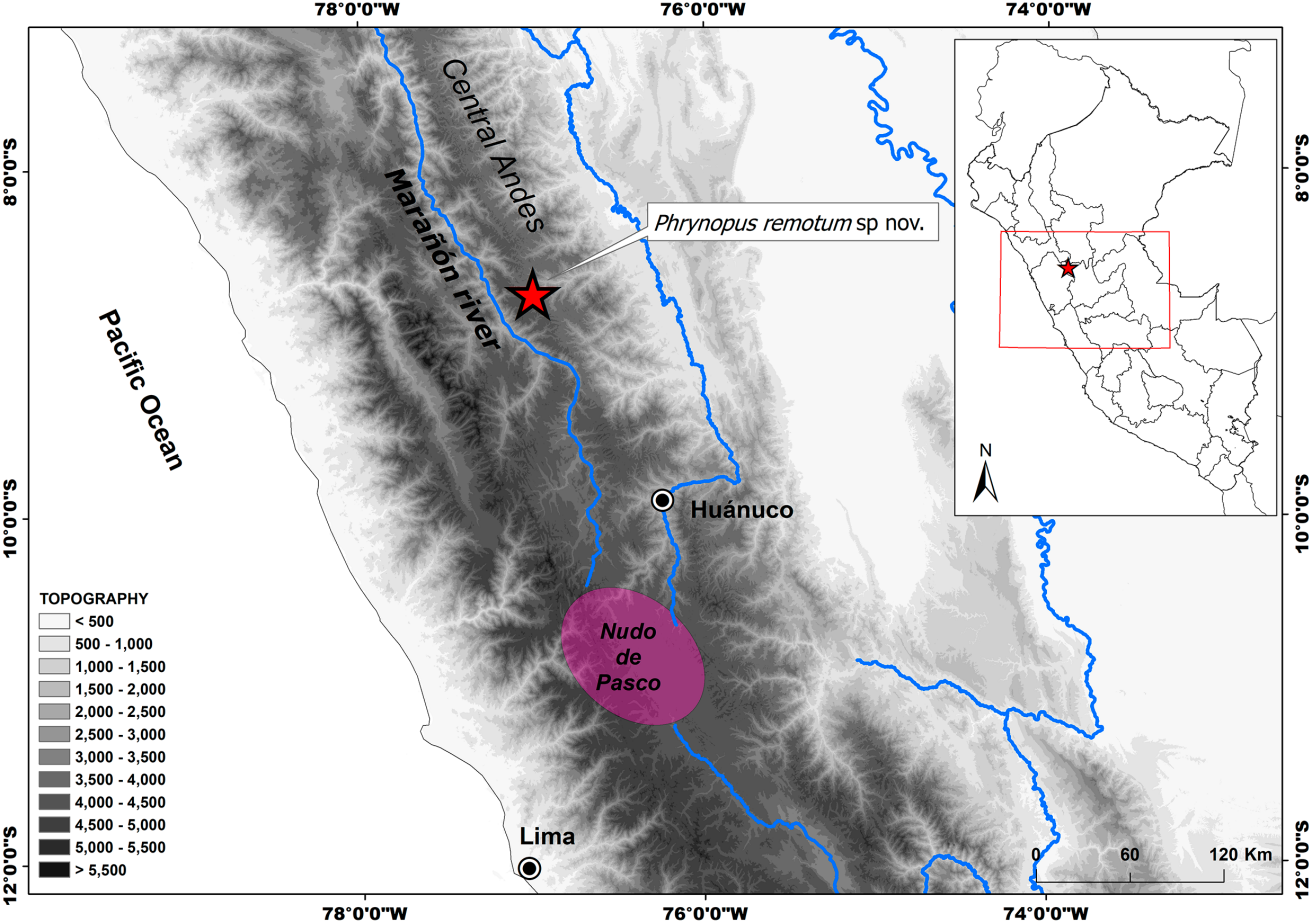
Distribution of *Phrynopus remotum.* sp nov. Map showing type locality (red star) of *Phrynopus remotum* sp. nov. and its location in central Peru, in the western slopes of the central Andes.

During an expedition to a remote locality in the Peruvian Central Andes, Departamento de Huánuco (see Fig. 1), we obtained a few specimens of *Phrynopus*. Detailed external revision of the specimens revealed unique combinations of morphological features not found in any other described species of *Phrynopus*. In addition to morphological comparisons, we used genetic data to examine the phylogenetic relationships of the new species. Here we present the results of our work and describe the new species.

## Material and Methods

We follow Lynch & Duellman (1997), Rodríguez & Catenazzi (2017) and Lehr et al. (2017) for format of description, as well as character definitions given by Duellman & Lehr (2009). We follow Hedges, Duellman & Heinicke (2008) and Heinicke et al. (2017) as support of our phylogenetic analyses for family placement. We collected specimens during the day while conducting opportunistic visual surveys. We euthanized specimens with benzocaine 20%, fixed them in 10% formalin, and stored them in 70% ethanol. Before fixation, we obtained tissue samples (liver) from all individuals and stored them in absolute ethanol. Specimens and tissue samples are deposited in the Herpetology Collection of Centro de Ornitología y Biodiversidad (CORBIDI). We used a digital caliper under a stereoscope to measure the following features to the nearest 0.1 mm: snout-vent length (SVL, from the tip of the nose to cloaca), tibia length (TL), foot length (FL, distance from proximal margin of inner metatarsal tubercle to tip of toe IV), head length (HL, from angle of jaw to tip of snout), head width (HW, at level of angle of jaw), eye diameter (ED), interorbital distance (IOD), upper eyelid width (EW), internarial distance (IND), and eye-to-nostril distance (E–N, straight line distance between anterior corner of orbit and posterior margin of external nares). Following Duellman & Lehr (2009), we numbered fingers and toes preaxially to post axially from I–IV and I–V respectively. We also determined comparative lengths of toes III and V by adpressing both toes against Toe IV; lengths of fingers I and II were determined by adpressing the fingers against each other (Duellman & Lehr, 2009). Specimens were sexed based on the examination of gonads after dissection of the ventral skin. We used high resolution photographs taken in the field to describe the coloration in life of the type series. We obtained information on species for comparative diagnoses from Duellman & Lehr (2009) and from original species descriptions. For detailed measurements of the type series see Table S1.

We conducted phylogenetic analyses to confirm generic placement of the new species, and to examine its evolutionary relationships with other species of *Phrynopus*. We relied on newly generated sequences from our specimens, the aligned sequences previously released by von May, Lehr & Rabosky (2018), and available sequences in GenBank for species of *Phrynopus* and related genera *Hypodactylus*, *Lynchius*, and *Oreobates* (as of 20 January 2020; Table S2; Padial, Grant & Frost, 2014; De la Riva et al., 2017; von May, Lehr & Rabosky, 2018). We analyzed fragments of five genes as previously described in von May, Lehr & Rabosky (2018), specifically including the three mitochondrial genes 12S, 16S, and the protein-coding gene cytochrome c oxidase subunit I (COI), and the two nuclear genes recombination-activating protein 1 (RAG1) and Tyrosinase precursor (Tyr). We followed standard protocols (Hedges, Duellman & Heinicke, 2008) for extraction, amplification, and sequencing of DNA, using the same primers and amplification protocols of von May, Lehr & Rabosky (2018). We ran the polymerase chain reaction (PCR) with a ProFlex thermal cycler (Applied Biosystems). We purified PCR products with Exosap-IT Express (Affymetrix, Santa Clara, CA), and shipped the purified products to MCLAB (South San Francisco, CA) for sequencing. We follow von May, Lehr & Rabosky (2018) and also used Geneious R11, version 11.1.5 (Biomatters, http://www.geneious.com/) to align the sequences with the MAFFT, version 7.017 alignment program (Katoh & Standley, 2013). We used MrBayes, version 3.2.0 (Ronquist & Huelsenbeck, 2003) for phylogenetic inference of the concatenated sequence of the five gene fragments. We used PartitionFinder software version 1.1.1 to select the best partitioning scheme and substitution model for each gene. Our analysis included 38 terminals and a 2,646-bp alignment for the partitioned dataset, branch length was unlinked among the partitions. We used the same partition scheme of von May, Lehr & Rabosky (2018), with six subsets as follows (each set corresponding to a number from 1 to 6): (1) model GTR + I + G for sequences 16S and 12S, (2) model K80 + I for 1st codon position of COI, (3) HKY + I for 2nd codon position of COI, (4) GTR + G for 3rd codon position of COI, (5) HKY + I for 1st and 2nd codon positions of RAG1 and Tyr, and (6) HKY + G for 3rd codon position of RAG1 and Tyr. We conducted the MCMC Bayesian analysis in MrBayes consisting of two simultaneous runs (each with three heated chains and one cold chain; burn-in set to discard the first 25% from the cold chain) of five million generations, with a sampling rate of once every 1,000 generations. The average standard deviation of split frequencies for the MCMC run was 0.00088. We used Geneious to display and export the majority-rule consensus tree along with the posterior probability values associated with each node.

Our research was approved by the Institutional Animal Care and Use Committee of Florida International University (18–009). We obtained our research permit through the Dirección General Forestal y de Fauna Silvestre, Ministerio de Agricultura y Riego, Peru, which issued the authorization 010-2018-SERFOR/DGGSPFFS, and the Contrato de Acceso Marco a Recursos Genéticos, numbered 359-2013-MINAGRI-DGFFS-DGEFFS.

The electronic version of this article will represent a publication, which agree with the International Commission on Zoological Nomenclature (ICZN), and hence the new names contained in the electronic version are effectively published under that Code from the electronic edition alone. This published work and the nomenclatural acts it contains have been registered in ZooBank, the online registration system for the ICZN. The ZooBank LSIDs (Life Science Identifiers) can be resolved and the associated information viewed through any standard web browser by appending the LSID to the prefix http://zoobank.org/. The LSID for this publication is: urn:lsid:zoobank.org:pub:382DA006-9656-4FB8-A617-66ECE8573CF4. The online version of this work is archived and available from the following digital repositories: PeerJ, PubMed Central, and CLOCKSS.

## Results

### Generic assignment

**Figure 2:**
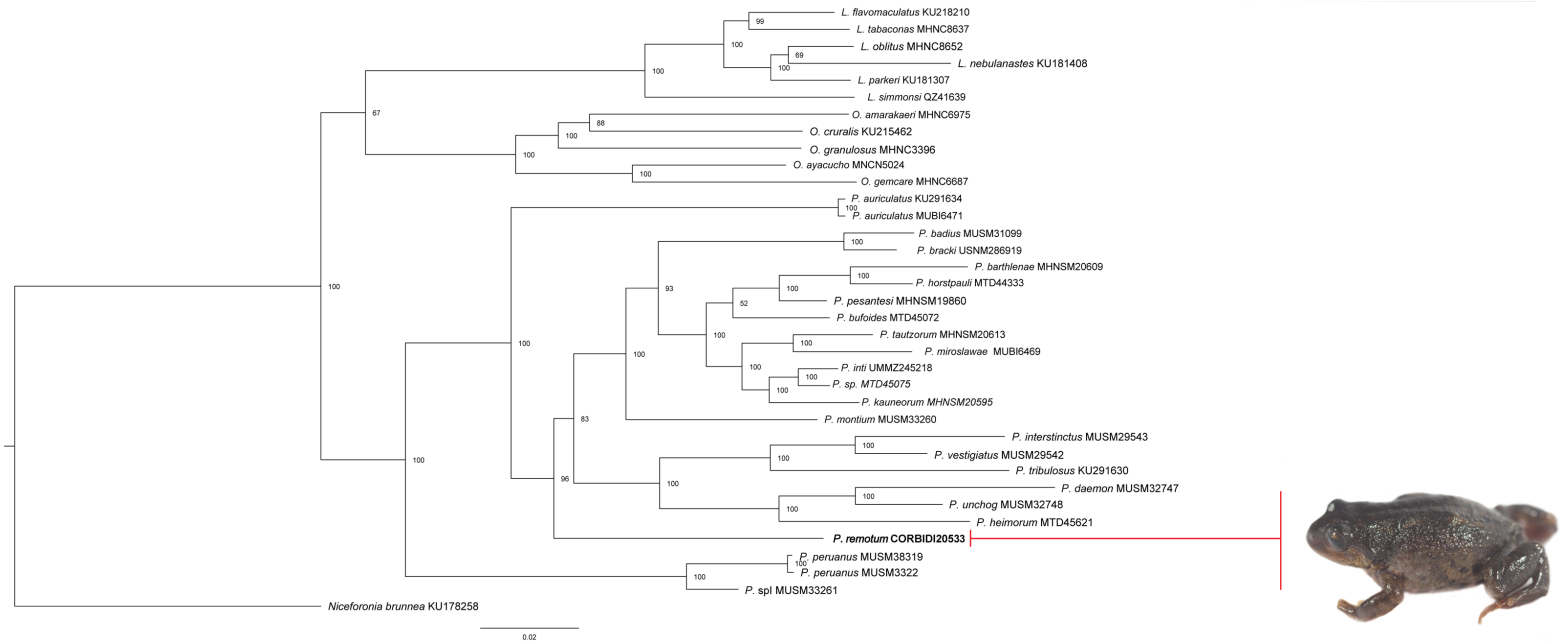
Phylogeny of *Phrynopus.* inferred with a Bayesian approach. Bayesian maximum clade-credibility tree for *Lynchius*, *Oreobates* and *Phrynopus* species included in this study based on a 2,646-bp concatenated dataset of gene fragment of 12S and 16S rRNA, cytochrome c oxidase subunit 1, recombination-activating protein 1, and tyrosinase precursor, analyzed in MrBayes (posterior probabilities are indicated at each node). The new species *P. remotum* is in bold.

The phylogeny inferred using the Bayesian approach supports the hypothesis that the new species is closely related to species in the genus *Phrynopus* (Fig. 2). Our inferred phylogeny closely matches the phylogeny of von May, Lehr & Rabosky (2018). Specifically, the new species is a sister taxon to most other species of *Phrynopus* except *P. auriculatus*, *P. peruanus* and an undescribed species (*P.* sp. “I” in von May, Lehr & Rabosky, 2018).

We currently lack evidence of any synapomorphic phenotypic trait for *Phrynopus* that allows distinguishing species in this genus from *Pristimantis* and other Terrarana with similar body shape and anatomical structures. These phenotypic features (referred to Phrynopoid ecomorph by De la Riva, 2020) presumably converged in response to the conditions of high elevation environments (Padial, Grant & Frost, 2014; Rodríguez & Catenazzi, 2017). Therefore, based on the results of our phylogenetic analyses, overall body shape and combination of meristic traits, we assign the new species to the genus *Phrynopus*.

### Species account

***Phrynopus*** ***remotum*** **sp. nov.** lsid:zoobank.org:act: 27E3AEAB-88C9-4D7A-88C8-E02A7E2794F9 http://zoobank.org/NomenclaturalActs/27e3aeab-88c9-4d7a-88c8-e02a7e2794f9

**Figure 3:**
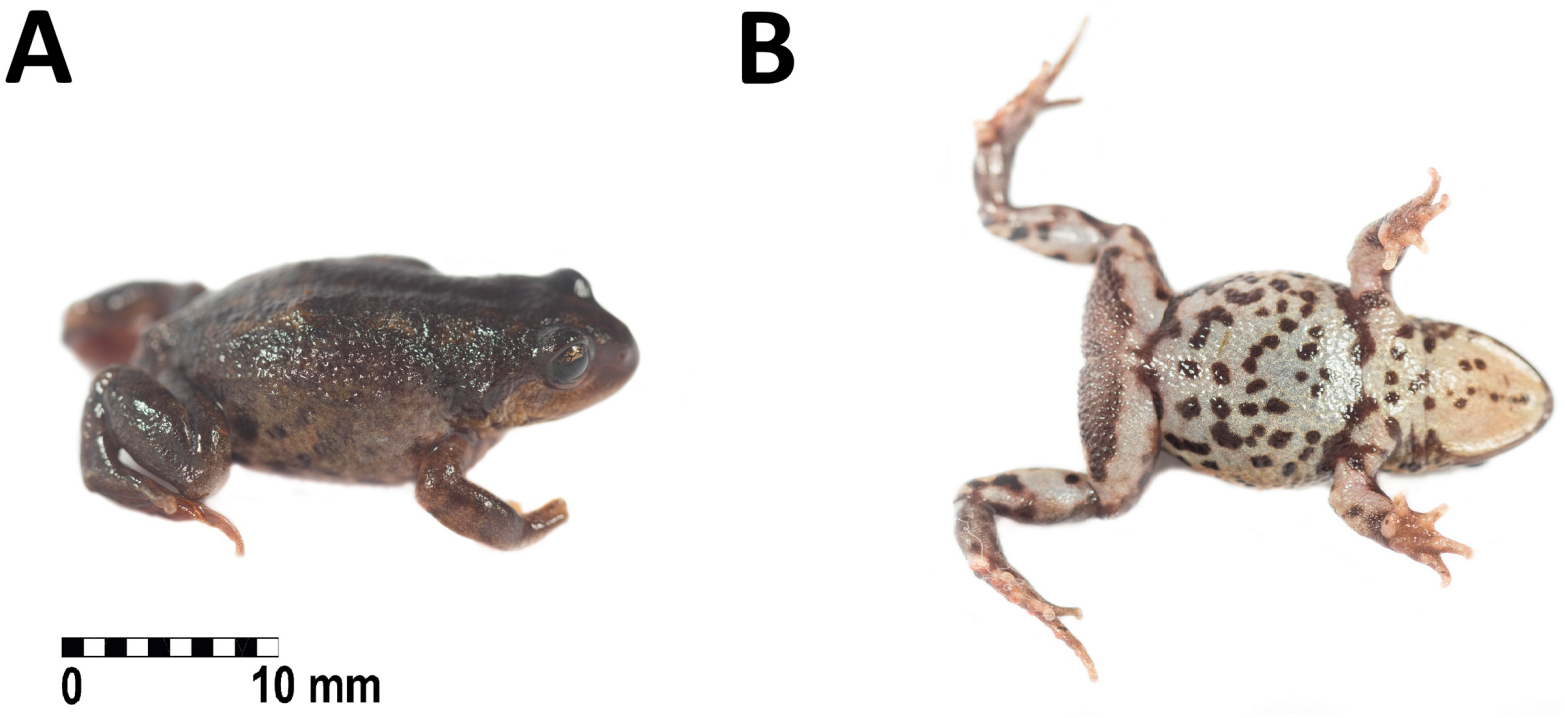
Holotype of *Phrynopus remotum.* sp nov. in life. Dorsal and ventral view of the holotype in life of *Phrynopus remotum* sp nov. (CORBIDI 20533, SVL =28.7 mm).

**Figure 4:**
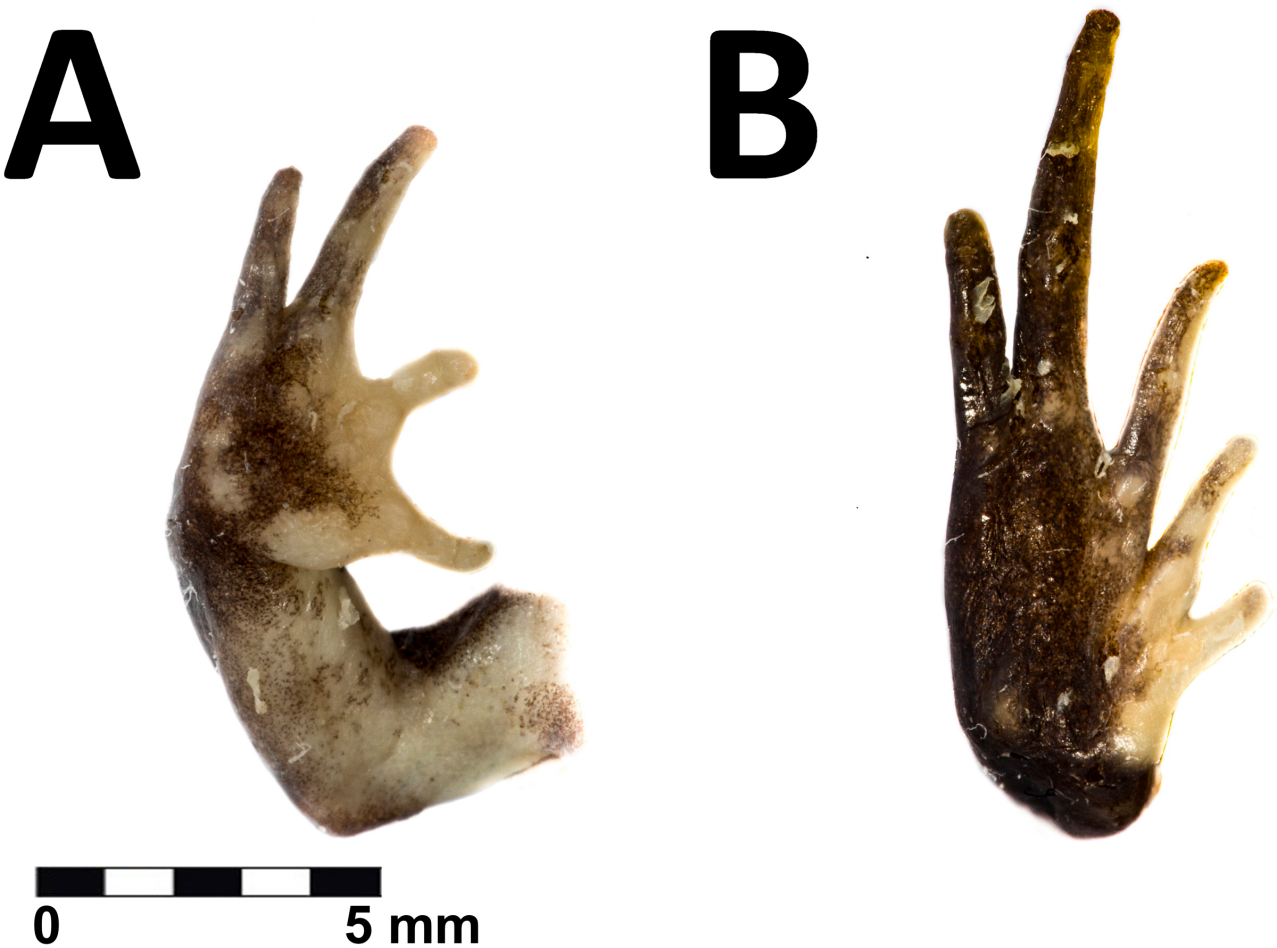
Hand and foot of the holotype of *Phrynopus remotum* sp nov. Ventral view of the hand (A) and foot (B) of the preserved holotype of *Phrynopus remotum* sp nov.

**Holotype.** Adult female (CORBIDI 20533, Figs. 3A–3B and 4A–4B) collected by GC near Villa Rica de Chona, on the trail to Antaquero Community (8°43′38.2″S, 76°59′25.95″W; 3,730 m a.s.l.), Marañón Province, Departamento de Huánuco, Peru on October 16th, 2018.

**Figure 5:**
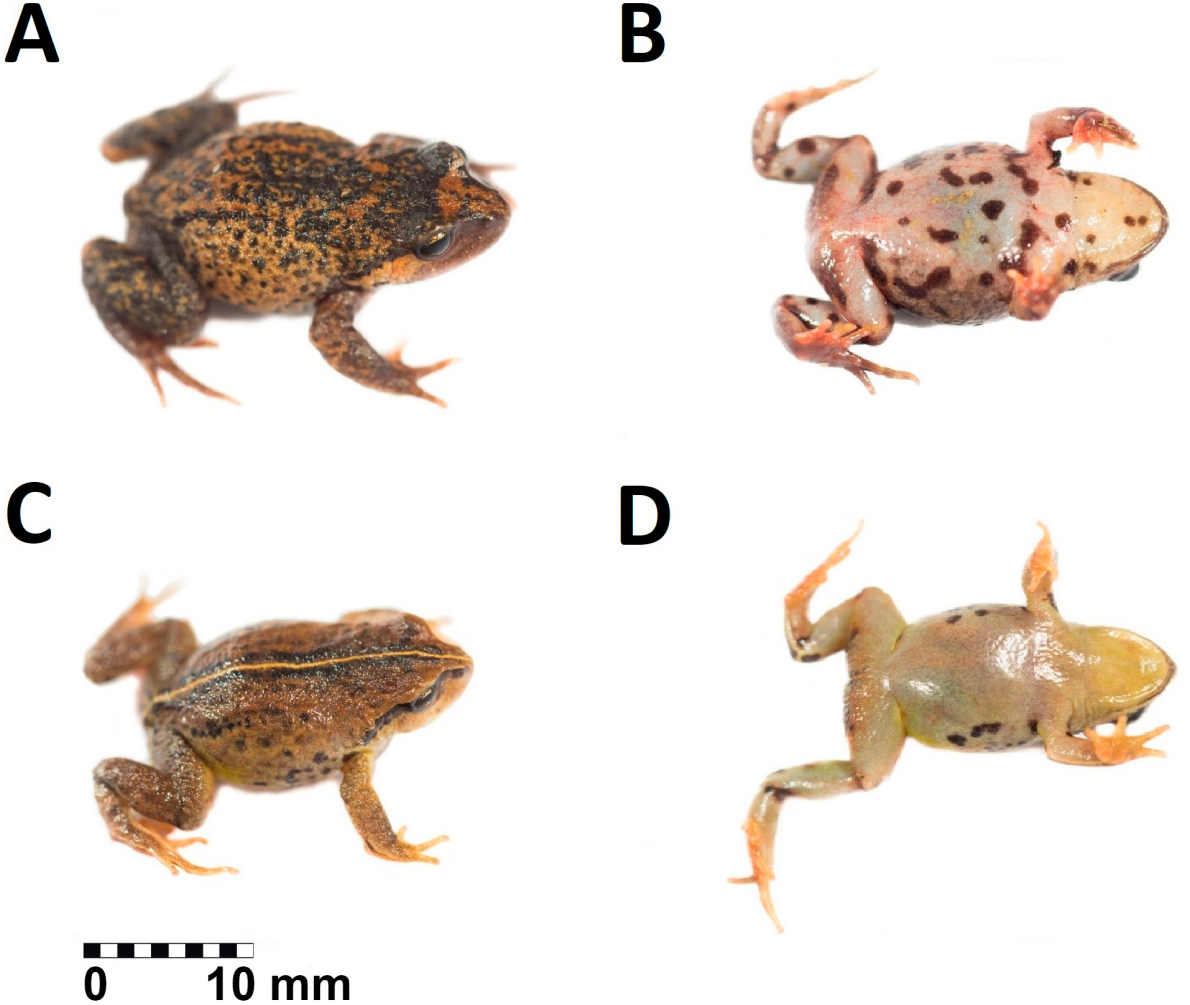
Paratypes of *Phrynopus remotum.* sp nov. in life. Dorsal (left column) and ventral (right column) views of the paratypes: (A–B) CORBIDI 20531 (SVL =23.3 mm); (C–D) CORBIDI 20532 (SVL =19.3 mm).

**Paratopotypes.** Adult males (CORBIDI 20531–32, Figs. 5A–5D) collected with the holotype.

### Diagnosis

A species of *Phrynopus* having the following combination of characters: (1) Skin on dorsum shagreened with scattered low subconical tubercles, skin on venter areolate; discoidal fold absent, thoracic fold absent; postocular fold absent, dorsolateral folds absent; (2) tympanic membrane and tympanic annulus absent; (3) snout short, rounded in dorsal view, curved anteroventrally from lateral view; (4) upper eyelid with small rounded tubercles; width of upper eyelid 1.9–2 mm; (5) dentigerous process of vomers present, oblique; (6) vocal slits and nuptial pads absent; (7) Finger I slightly shorter than Finger II; tips of digit bulbous, rounded, lacking discs; (8) fingers without lateral fringes; (9) ulnar and tarsal tubercles absent; (10) heels with one or two small rounded tubercles, inner tarsal fold absent; (11) inner metatarsal tubercle rounded, about 1.3 times as large as ovoid outer metatarsal tubercle; supernumerary plantar tubercles absent; (12) toes without lateral fringes, basal webbing absent, Toe V slightly shorter than Toe III, toe tips bulbous, lacking discs; (13) in life, dorsum saffron yellow, brownish-orange or dark brown with dark brown or black blotches, dark spots on flanks; throat, chest and venter yellowish-white with dark brown blotches or spots, groin grayish-white with liver brown or dark brown spots and blotches; iris olive gray with fine dark brown reticulations; (14) SVL 19.3 and 23.3 mm in two males, and 28.7 mm in a single female.

**Comparisons.-** The absence of tympanic membrane and tympanic annulus distinguishes *Phrynopus remotum* sp. nov. from *P. auriculatus*, *P. mariellaleo* and *P. peruanus*. The new species has a shagreened dorsum, differentiating it from *P. anancites* (coarsely areolate), *P. miroslawae* (coarsely tuberculate), *P. juninensis*, *P. kauneorum*, and *P. tautzorum* (smooth), and *P. bracki*, *P. bufoides*, *P. capitalis*, *P. dagmarae*, *P. heimorum*, *P. horstpauli*, and *P. lapidoides* (tuberculate). Furthermore, *P. remotum* is clearly different from *P. inti*, *P. tribulosus*, and *P. valquii* by having an areolate venter (smooth in all of them). The absence of dorsolateral folds or longitudinal dorsal ridges makes *P. remotum* sp. nov. distinct from *P. badius*, *P. daemon*, *P. dumicola*, *P. interstinctus*, *P. kotosh*, *P. paucari*, *P. personatus*, *P. thompsoni*, *P. unchog*, and *P. vestigiatus*. *Phrynopus remotum* is also distinguishable from *P. barthlenae*, *P. chaparroi*, *P. montium*, *P. oblivious*, and *P. pesantesi* by bearing of dentigerous process of vomers. Finally, *P. remotum* sp. nov. differs from *P. lechriorhynchus* by being larger with males reaching up 23.3 m.m (16.6 mm) and of low rounded tubercles on upper eyelid and heels (absent).

**Description of the holotype.-** an adult female (CORBIDI 20533), head as wide as body, wider than long; head width is 126% of head length; head width is 36% of snout-vent length; head length is 28% of snout-vent length. The snout is short, rounded in dorsal view, curved anteroventrally in lateral view (Figs. 3A–3B), eye-to-nostril distance is 83% of eye diameter; nostrils are protuberant, directed dorsolaterally; *canthus rostralis* is slightly curved in dorsal view, rounded in profile; loreal region slightly concave; lips rounded; upper eyelid with small rounded tubercles, narrower than interorbital distance (EW 71% of IOD); postocular and tympanic region without folds (Fig. 3A); tympanic membrane and tympanic annulus absent, tympanic region lacking of postrictal tubercles. The choanae are small, ovoid, close to palatal shelf of maxilla; dentigerous processes of vomers present, oblique, located posterior to the level of choanae and broadly separated from each other; tongue broad, about 1.5 as long as wide, not notched posteriorly, posterior half free; vocal slits absent.

The skin on dorsum is shagreened, and has rounded and apical scattered tubercles (Fig. 3A); skin on flanks shagreened with scattered rounded tubercles; skin on throat, chest and belly areolate (Fig. 3B); discoidal fold absent, thoracic fold present; cloacal sheath not distinct; cloacal region with high rounded tubercles. Outer surface of forearm without tubercles; outer palmar tubercle barely visible, low, ovoid, smaller than ovoid inner palmar tubercle; supernumerary tubercles absent; subarticular tubercles low, ovoid, most prominent on base of fingers; fingers without lateral fringes; Finger I slightly shorter than Finger II (Fig. 4A); tips of digits rounded, bulbous, lacking circumferential grooves.

The hind limbs are long and slender, tibia length is 34% of the snout-vent length; foot length is 38% of the snout-vent length; dorsal surface of hind limbs shagreened; anterior surfaces of thighs shagreened, posterior surfaces of thighs tuberculate; heel with low rounded tubercles; outer surface of tarsus without tubercles; outer metatarsal tubercle rounded and prominent, about same size as prominent ovoid inner metatarsal tubercle; supernumerary plantar tubercles absent; subarticular tubercles low, ovoid in dorsal view, most distinct on the base of toes; toes without lateral fringes; basal webbing absent; toe tips bulbous, rounded, lacking circumferential grooves, about as large as those on fingers; relative lengths of toes: 1 <2 <3 <5 <4; Toe V slightly shorter than Toe III (Fig. 4B).

**Measurements of the holotype (in mm).** SVL 28.7; tibia length 9.7; foot length 11.0; head length 8.2; head width 10.4; eye diameter 2.8; interorbital distance 2.8; upper eyelid width 2.0; internarial distance 2.5; eye-nostril distance 1.9.

**Coloration of the holotype in life (Figs. 3A–3B).** Dorsum dark brown with some pale areas on posterior third, flanks brownish-yellow with dark brown blotches. Postocular stripe black. Upper lip saffron yellow. Arms and legs dorsally yellowish-brown. Throat yellowish-white with dark brown spots on the distal edge, chest and belly creamy white with dark brown blotches on the region nearest to the insertion of forelimbs and mixed with dark brown spots on the rest of venter. Groin dark brown with grayish-white reticulations, posterior surfaces of thighs dark brown, posterior surfaces of tibias and dorsal surfaces of feet yellowish-brown. Iris olive yellow with dark brown reticulations.

**Coloration of the holotype in preservative** Dorsum and flanks dark brown with some pale scattered areas. Postocular stripe dark brown. Upper lip tan. Arms and legs dorsally dark brown. Throat creamy white with pale brown spots on the distal edge, chest and belly creamy white with pale brown blotches on the region nearest to the insertion of forelimbs and mixed with brown spots on the rest of venter. Groin pale brown with creamy white reticulations, posterior surfaces of thighs tan, posterior surfaces of tibias and dorsal surfaces of feet brown. Iris bluish-yellow.

**Variation.** All paratypes (Figs. 5A–5D) are morphologically similar to the holotype (see Table S1 for variation in measurements and proportions), but some differ in coloration. General color patterns range from saffron yellow in male CORBIDI 20532 (Figs. 5C–5D), to yellowish-orange in male CORBIDI 20531 (Figs. 5A–5B). CORBIDI 20531 has a longitudinal yellow black bordered stripe extending from the tip of the nose to the edge of the anal region. The canthal region is brown in the male CORBIDI 20532, and yellow in male CORBIDI 20531. The ventral coloration is grayish-yellow without dark blotches and bearing dark spots at the lateral edges of belly, in the male CORBIDI 20531.

**Etymology.** The specific name *remotum* is the neutral form derived from the Latin word “remotus”, in reference to the long journey required to reach the type locality of this species. This journey consisted of more than 30 h traveling through roadways, hiking trails and steep slopes of rocky mountains.

**Figure 6:**
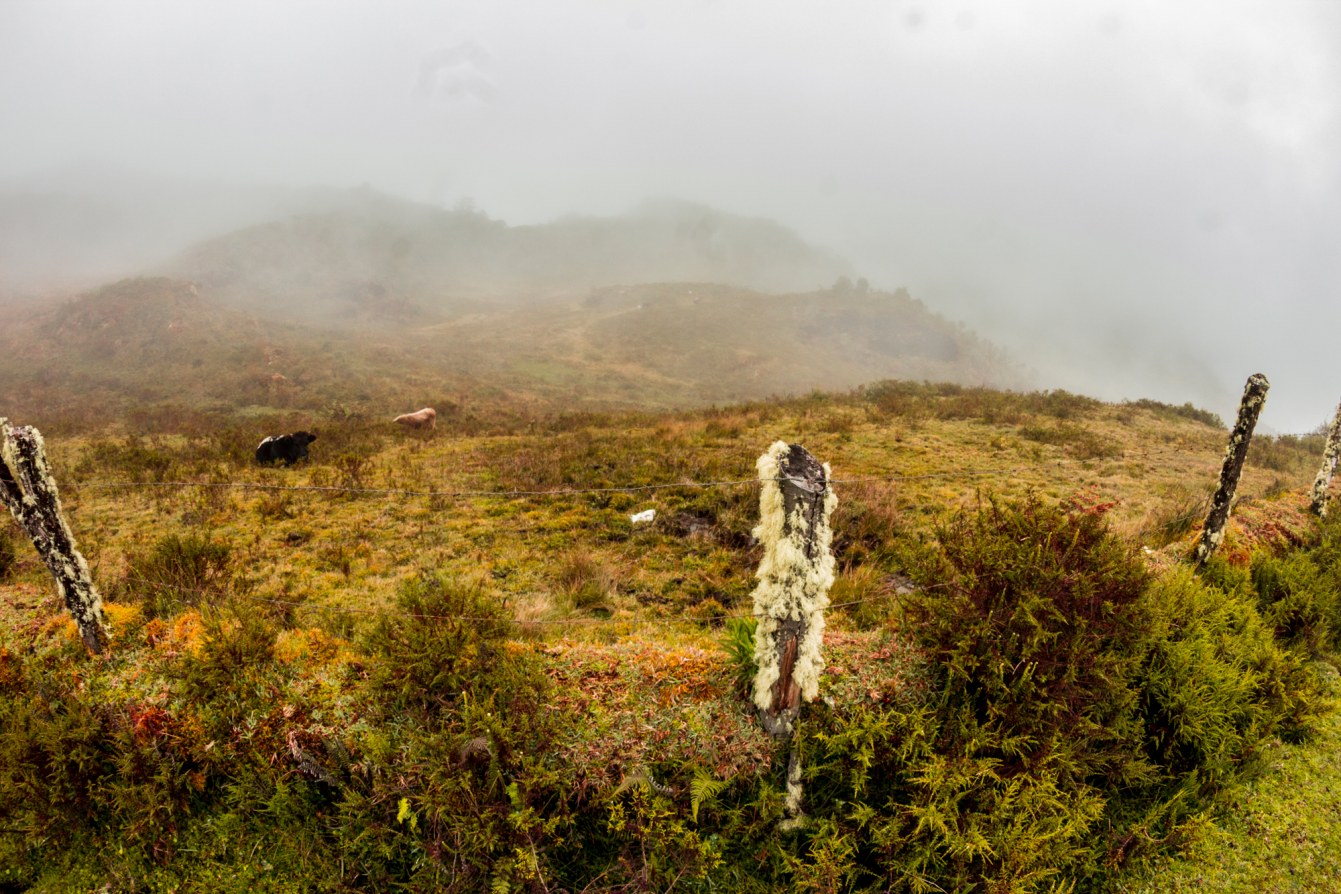
Habitat of *Phrynopus remotum.* sp nov. in the Puna grasslands of the central Pruvian Andes (Huánuco Department).

**Distribution, natural history, and conservation status.** This species is known only from the type locality at 3,700 m a.s.l. in the eastern Andean slopes of Central Peru (Fig. 1). The habitat is a transitional area between wet grasslands and some remaining patches of cloud forest and bushes. We found all specimens around 12:30 pm under stones surrounded by moss and lichens, alongside a temporary stream bordering a wet grassland. The only sympatric amphibian found was *Gastrotheca peruana*, which was frequently spotted under stones and also beneath the moss; however none of these frogs were found sharing a stone with *P. remotum* sp. nov. We noticed heavy presence of livestock (Fig. 6). Local people annually burn the grassland to trigger regrowth of grass plants (*Festuca*, *Stipa* spp.) and to open new pasture grounds for cattle. Despite these threats (Catenazzi & von May, 2014), the paucity of data on the geographic distribution of this species prevents assessment of its threat status. Therefore, we recommend the Category Data Deficient for the IUCN Red List. Future surveys should document the range of the species and assess the importance of threats.

## Discussion

Similar to most congeneric species, *Phrynopus remotum* inhabits humid puna grasslands in the Peruvian Andes (Lehr, Fritzsch & Müller, 2005; Chávez et al., 2015; Lehr & Rodriguez, 2017; Rodríguez & Catenazzi, 2017). Several *Phrynopus* species have been reported from two or more localities (Trueb & Lehr, 2008; von May, Lehr & Rabosky, 2018), but so far *P. remotum* is only known from the type locality; however, we are confident that future exploration in surrounding areas could detect the new species, and contribute to better documenting its distribution range.

Geographical barriers could be the main factor explaining diversification and distribution of vertebrates in Andean ecosystems (Winger & Bates, 2015; Winger, 2017; Hazzi et al., 2018). The type locality of *Phrynopus remotum* lies in the upper Marañón basin (Peruvian Central Andes), an arid valley previously hypothesized as an important geographic feature for amphibians, birds, and plants (Sarkinen et al., 2010; Hazzi et al., 2018). The adjacent mountains, characterized by a rugged topography and lack of roads, are very hard to reach, and that is why they are still poorly explored. Therefore, further field surveys in the highlands will likely result in the discovery of additional species of *Phrynopus*, which will help us understand their biology and spatial distribution. The Nudo de Pasco, an elevated Andean ridge resulting from the convergence of the Western, Central and Eastern ridges of the Andes (Fig. 1), is a major physical feature south of the Marañón dry Valley and is also where this river originates. The highest diversity of *Phrynopus* is concentrated in humid grasslands and montane forests in this region. The aridity of the Marañón dry Valley likely contributed as a geographical barrier promoting isolation and subsequent species diversification (Winger & Bates, 2015; Winger, 2017; Hazzi et al., 2018). Whereas the aridity of the upper Marañón Valley likely limits the distribution of *Phrynopus* species, the higher moisture in the high-elevations of the Nudo de Pasco should have promoted their dispersal and distribution. Thus, further studies should focus on the diversification and distribution patterns of *Phrynopus* in the Nudo de Pasco region.

Given their restricted geographical range, their limited dispersal abilities, and the increasing livestock and agriculture in the area (Finer, Novoa & Snelgrove, 2015), *Phrynopus* frogs could face serious threats such as habitat loss and degradation or grassland fires. These similar to threats faced by other *Phrynopus* species in the Peruvian Andes (Catenazzi & von May, 2014; Lehr et al., 2017). Thus, field data on occurrence and habitat use of *Phrynopus* are critical to propose conservation initiatives benefiting these frogs. Future field efforts should document species distribution and collect data that allow stakeholders to develop relevant conservation plans.

## Conclusions

We present genetic and phenotypic evidence which support the description of *Phrynopus remotum* sp. nov. This species has a very narrow distribution range, and is only known from three individuals collected at the type locality. However, due to the scarce fieldwork in the area, we cannot assume that its distribution is restricted to a single locality, thus further surveys are needed. Additionally, this discovery highlights the importance of protecting the Marañón dry Valley and adjacent highlands, threatened by the rapid expansion of human activities.

##  Supplemental Information

10.7717/peerj.9433/supp-1 Data S1 Raw measurements of the type series of Phrynopus remotum sp nov

10.7717/peerj.9433/supp-2 Table S1 Measurements of the type series of *Phrynopus remotum* Range of measured characters (in mm) and proportions of *Phrynopus remotum* sp nov. Range of measured characters is followed by mean value and one standard deviation.

10.7717/peerj.9433/supp-3 Table S2 Accession codes of GenBank of the sequences used in this study GenBank accession numbers for the taxa and genes sampled in this study. New sequences produced for this study (*P. remotum*, CORBIDI 20531) are in bold.

10.7717/peerj.9433/supp-4 Supplemental Information 4 DNA sequences_P_remotum

## Additional Information and Declarations

### Field Study Permissions

The following information was supplied relating to field study approvals (i.e., approving body and any reference numbers): The Dirección General Forestal y de Fauna Silvestre, Ministerio de Agricultura y Riego of Peru issued the permit authorizing this research (collecting permit 010-2018-SERFOR/DGGSPFFS, Contrato de Acceso Marco a Recursos Genéticos, No 359-2013-MINAGRI-DGFFS-DGEFFS).

### DNA Deposition

The following information was supplied regarding the deposition of DNA sequences: The sequences of the new species are available at GenBank: MT261773–MT261774, MT261899, MT263073, MT272829, MT431667–MT431671, MT434009–MT434010.

### Data Availability

The following information was supplied regarding data availability: The raw data of the measurements and the genetic sequences are available in the Supplemental Files. The sequences are also available at GenBank: MT261773–MT261774, MT261899, MT263073, MT272829, MT431667–MT431671, MT434009–MT434010. Our specimens (CORBIDI 20531, 20532, 20533) are stored in the Division de Herpetologia of CORBIDI, Lima, Peru.

### New Species Registration

The following information was supplied regarding the registration of a newly described species: Publication LSID: urn:lsid:zoobank.org:pub:382DA006-9656-4FB8-A617-66ECE8573CF4 Phrynopus LSID: urn:lsid:zoobank.org:act:6219BEA7-16DA-4438-BB2D-E88024428601 *Phrynopus remotum* sp. nov. LSID: urn:lsid:zoobank.org:act:27E3AEAB-88C9-4D7A-88C8-E02A7E2794F9.
