# Supplementary material for: A new species of frog (Terrarana, Strabomantidae, Phrynopus) from the Peruvian Andean grasslands
Source: PeerJ. 2020 Jun 24;8:e9433. doi: 10.7717/peerj.9433 (PMC7320723; doi:10.7717/peerj.9433)
Supplement: Data S1 [file peerj-08-9433-s001.docx]

Raw data measurements of the type series of Phrynopus remotum sp nov.

|  | **CORBIDI 20531 (Male)** | **CORBIDI 20532 (Male)** | **CORBIDI 20533 (Female)** |
| --- | --- | --- | --- |
| SVL | 19.3 | 23.3 | 28,7 |
| HL | 5.4 | 7.5 | 8,2 |
| HW | 7.4 | 9.3 | 10,4 |
| IOD | 1.8 | 2.1 | 2,8 |
| UEW | 1.6 | 1.9 | 2,0 |
| Internarial Distance | 1.9 | 2.4 | 2,5 |
| Eye to nostril | 1.5 | 1.8 | 1,9 |
| Eye to tip of the nose | 2.9 | 3.6 | 3,8 |
| Eye diameter | 1.7 | 2.1 | 2,8 |
| Femur length | 7.9 | 8.6 | 9,9 |
| Tibia length | 7.2 | 8.9 | 9,7 |
| Hand length | 4.8 | 5.2 | 6,5 |
| Foot length | 8.1 | 9.5 | 11,0 |
| Fourth finger length | 1.8 | 2.5 | 2,4 |
| Fourth toe length | 4.7 | 5.8 | 6,1 |
| fourth toe width | 0.5 | 0.7 | 0,7 |
| HL/SVL | 0.2 | 0.3 | 0,2 |
| HW/SVL | 0.3 | 0.4 | 0,3 |
| HW/HL | 1.2 | 1.3 | 1,2 |
| UEW/IOD | 0.8 | 0.8 | 0,7 |
| E-N/ED | 0.8 | 0.8 | 0,6 |
| TL/SVL | 0.3 | 0.3 | 0,3 |
| FL/SVL | 0.4 | 0.4 | 0,3 |
