# Supplementary material for: A new species of frog (Terrarana, Strabomantidae, Phrynopus) from the Peruvian Andean grasslands
Source: PeerJ. 2020 Jun 24;8:e9433. doi: 10.7717/peerj.9433 (PMC7320723; doi:10.7717/peerj.9433)
Supplement: Table S1 — Range of measured characters (in mm) and proportions of Phrynopus remotum sp nov. Range of measured characters is followed by mean value and one standard deviation. [file peerj-08-9433-s002.docx]

**Table S1.** Range of measured characters (in mm) and proportions of *Phrynopus remotum* sp. nov. Range of measured characters is followed by mean and standard deviation.

|  | *Phrynopus remotum sp nov.* | |
| --- | --- | --- |
|  | Male n=2 | Female n=1 |
| SVL | 19.3-23.3 (21.3 ± 2.8) | 28.7 |
| HL | 5.4-7.5 (6.5 ± 1.4) | 8.2 |
| HW | 7.4-9.3 (8.4 ± 1.3) | 10.4 |
| IOD | 1.8-2.1 (2.0 ± 0.1) | 2.8 |
| EW | 1.6-1.9 (1.8 ± 0.1) | 2.0 |
| IND | 1.9-2.4 (2.1 ± 0.3) | 2.5 |
| E−N | 1.5-1.8 (1.7 ± 0.1) | 1.9 |
| Eye to tip of the nose | 2.9-3.6 (3.2 ± 0.4) | 3.8 |
| Eye diameter | 1.7-2.1 (1.9 ± 0.2) | 2.8 |
| Tibia length | 7.2-8.9 (8.1 ± 1.2) | 9.7 |
| Foot length | 8.1-9.5 (8.8 ± 0.9) | 11.0 |
| Fourth finger length | 1.8-2.5 (2.1 ± 0.4) | 2.4 |
| Fourth toe length | 4.7-5.8 (5.2 ± 0.7) | 6.1 |
| fourth toe width | 0.5-0.7 (0.6 ± 0.1) | 0.7 |
| HL/SVL | 0.2-0.3 (0.3 ± 0.0) | 0.2 |
| HW/SVL | 0.3-0.4 (0.3 ± 0.0) | 0.3 |
| HW/HL | 1.2-1.3 (1.3 ± 0.0) | 1.2 |
| EW/IOD | 0.8-0.8 (0.8 ± 0.0) | 0.7 |
| E−N/ED | 0.8-0.8 (0.8 ± 0.0) | 0.6 |
| TL/SVL | 0.3-0.3 (0.3 ± 0.0) | 0.3 |
| FL/SVL | 0.4-0.4 (0.4 ± 0.0) | 0.3 |
