# Supplementary material for: A new species of frog (Terrarana, Strabomantidae, Phrynopus) from the Peruvian Andean grasslands
Source: PeerJ. 2020 Jun 24;8:e9433. doi: 10.7717/peerj.9433 (PMC7320723; doi:10.7717/peerj.9433)
Supplement: Table S2 — GenBank accession numbers for the taxa and genes sampled in this study. New sequences produced for this study (P. remotum, CORBIDI 20531) are in bold. [file peerj-08-9433-s003.docx]

**Table S2.** GenBank accession numbers for the taxa and genes sampled in this study. New sequences produced for this study (*P. remotum*, CORBIDI 20531—33) are in bold.

| **Taxon** | **16S** | **12S** | **COI** | **RAG1** | **Tyr** | **Voucher #** |
| --- | --- | --- | --- | --- | --- | --- |
| *Hypodactylus brunneus* | EF493357 | EF493357 | na | EF493422 | EF493484 | KU178258 |
| *Lynchius flavomaculatus* | EU186667 | EU186667 | na | EU186745 | EU186766 | KU218210 |
| *Lynchius nebulanastes* | EU186704 | EU186704 | na | na | na | KU181408 |
| *Lynchius oblitus* | KX470782 | KX470775 | na | KX470791 | na | MHNC8652 |
| *Lynchius parkeri* | EU186705 | EU186705 | na | na | na | KU181307 |
| *Lynchius simmonsi* | JF810004 | JF809940 | na | JF809915 | JF809894 | QZ41639 |
| *Lynchius tabaconas* | KX470780 | KX470773 | na | na | KX470796 | MHNC8637 |
| *Oreobates amarakaeri* | JF809996 | JF809934 | na | JF809913 | JF809891 | MHNC6975 |
| *Oreobates ayacucho* | JF809970 | JF809933 | na | JF809912 | JF809890 | MNCN_IDlR5024 |
| *Oreobates cruralis* | EU186666 | EU186666 | na | EU186743 | EU186764 | KU215462 |
| *Oreobates gemcare* | JF809960 | JF809930 | na | JF809909 | na | MHNC6687 |
| *Oreobates granulosus* | EU368897 | JF809929 | na | JF809908 | JF809887 | MHNC3396 |
| *Phrynopus auriculatus* | EF493708 | EF493708 | na | na | na | KU291634 |
| *Phrynopus auriculatus* | MF186348 | MF186290 | MF186466 | na | MF186582 | MUBI 6471 |
| *Phrynopus badius* | MG896572 | MG896595 | MG896612 | MG896619 | na | MUSM31099 |
| *Phrynopus barthlenae* | MF186350 | MF186292 | MF186464 | na | na | MHNSM20609 |
| *Phrynopus bracki* | EF493709 | EF493709 | na | EF493421 | na | USNM286919 |
| *Phrynopus bufoides* | AM039645 | AM039713 | na | na | na | MTD45072 |
| *Phrynopus daemon* | MG896574 | MG896597 | na | na | na | MUSM32747 |
| *Phrynopus heimorum* | AM039635 | AM039703 | MF186462 | MF186545 | MF186580 | MTD45621 |
| *Phrynopus horstpauli* | MF186364 | MF186303 | na | na | MF186584 | MTD44335 |
| *Phrynopus interstinctus* | MG896575 | MG896598 | MG896614 | MG896621 | na | MUSM29543 |
| *Phrynopus inti* | MF651906 | MF651913 | na | MF651918 | MF651921 | UMMZ245218 |
| *Phrynopus juninensis* | MF651908 | MF651915 | na | MF651920 | na | MUSM33258 |
| *Phrynopus kauneorum* | AM039655 | AM039723 | na | na | na | MHNSM20595 |
| *Phrynopus miroslawae* | MF186393 | MF186312 | MF186463 | MF186542 | MF186585 | MUBI 6469 |
| *Phrynopus montium* | MG896579 | MG896602 | na | MG896625 | na | MUSM33260 |
| *Phrynopus peruanus* | MG896582 | MG896605 | MG896615 | MG896626 | MG896631 | MUSM38316 |
| *Phrynopus pesantesi* | AM039656 | AM039724 | na | na | na | MHNSM19860 |
| *Phrynopus remotum* | **MT261899** | na | **MT263073** | **MT431670** | **MT431667** | CORBIDI20531 |
| *Phrynopus remotum* | **MT261774** | na | **MT434010** | na | **MT431668** | CORBIDI20532 |
| *Phrynopus remotum* | **MT261773** | **MT272829** | **MT434009** | **MT431671** | **MT431669** | CORBIDI20533 |
| *Phrynopus* sp | AM039657 | AM039725 | na | na | na | MTD45075 |
| *Phrynopus* spI | MG896589 | MG896606 | na | MG896629 | na | MUSM33261 |
| *Phrynopus tautzorum* | AM039652 | AM039720 | na | na | na | MHNSM20613 |
| *Phrynopus tribulosus* | MF186424 | MF186330 | MF186467 | MF186547 | MF186579 | MUBI 7166 |
| *Phrynopus unchog* | MG896591 | MG896608 | na | na | na | MUSM32748 |
| *Phrynopus vestigiatus* | MG896593 | MG896610 | MG896617 | na | na | MUSM29542 |
